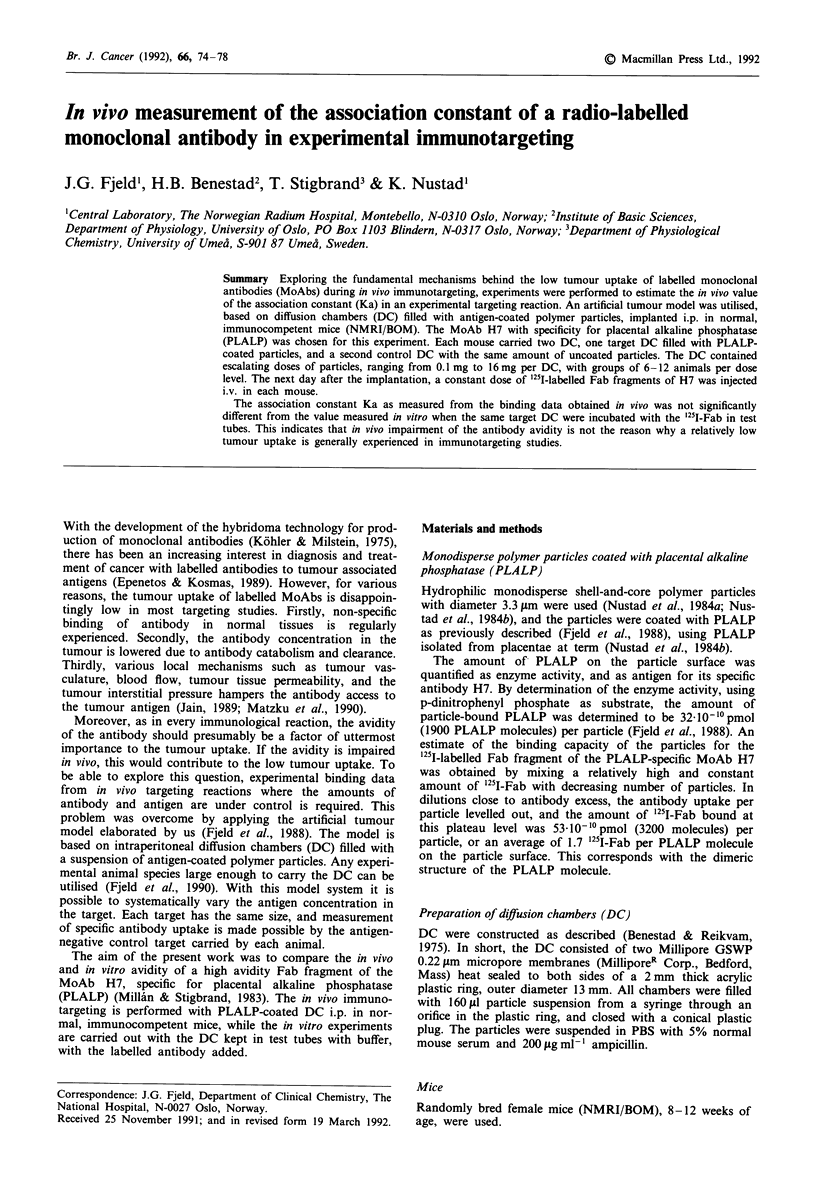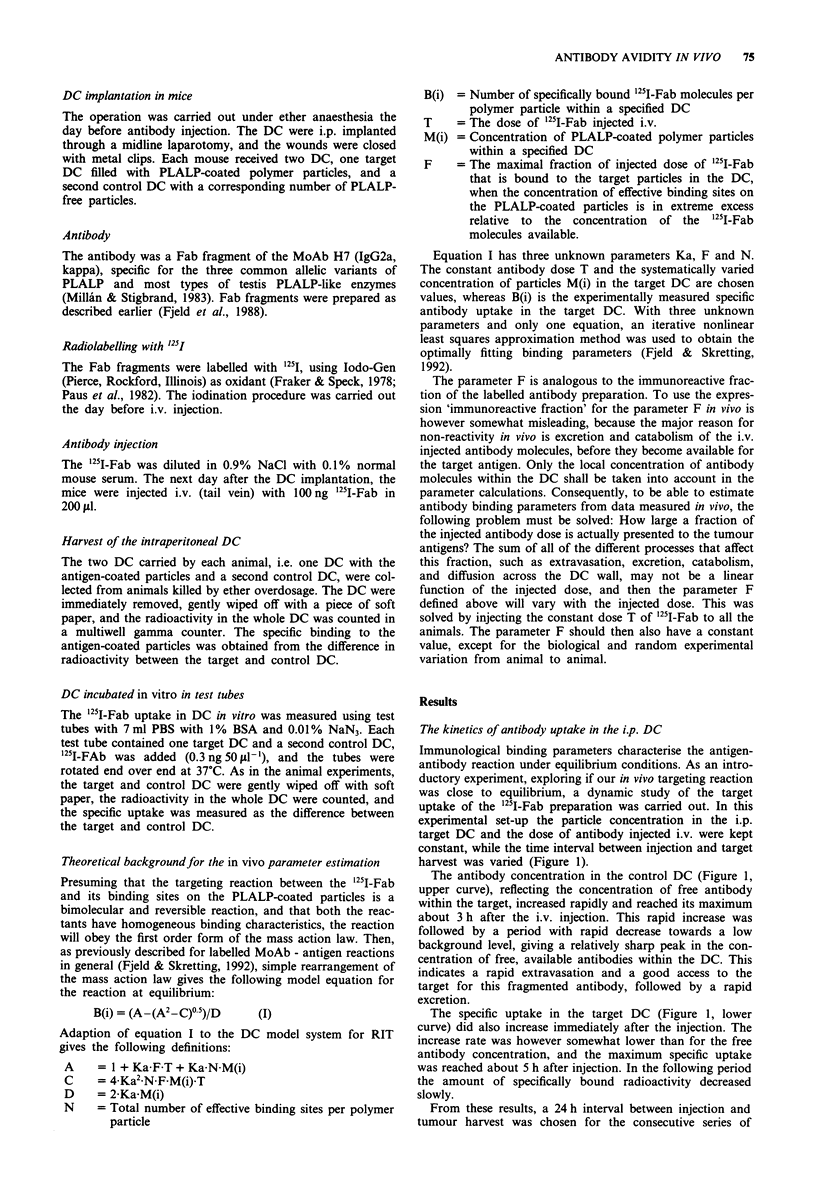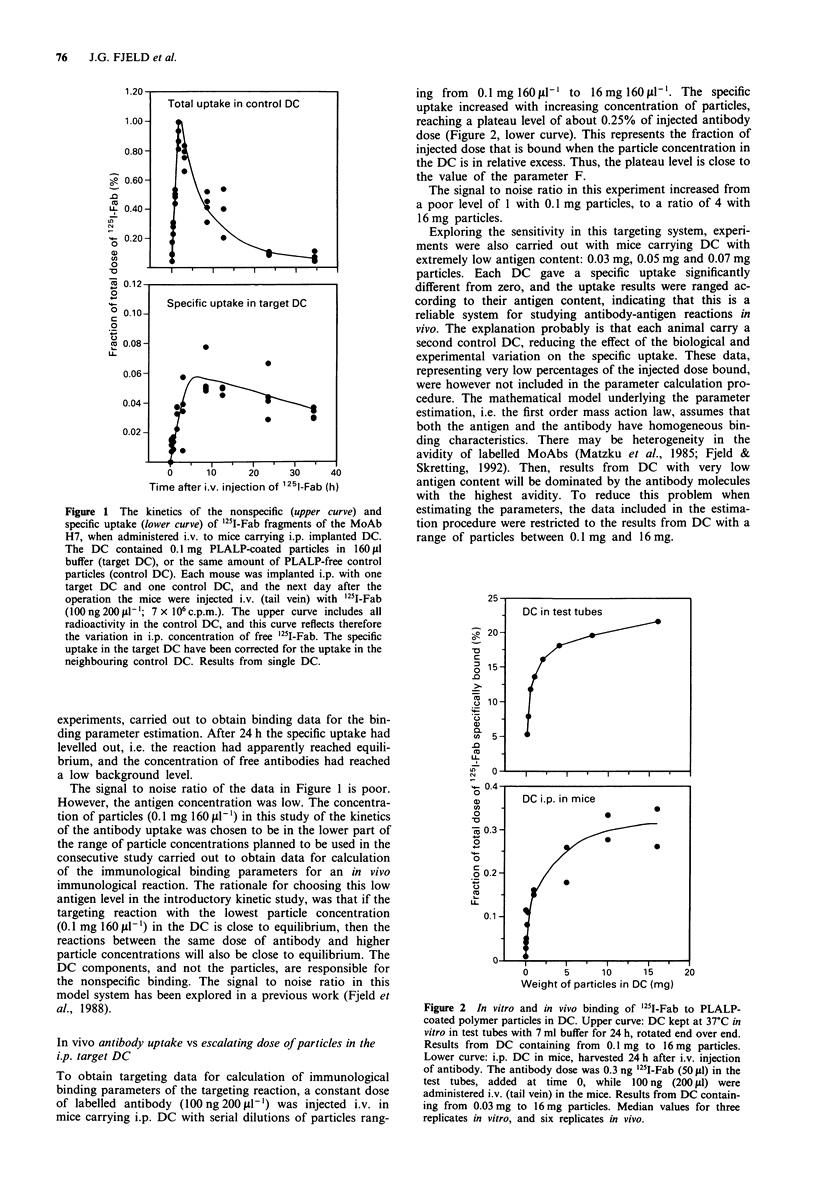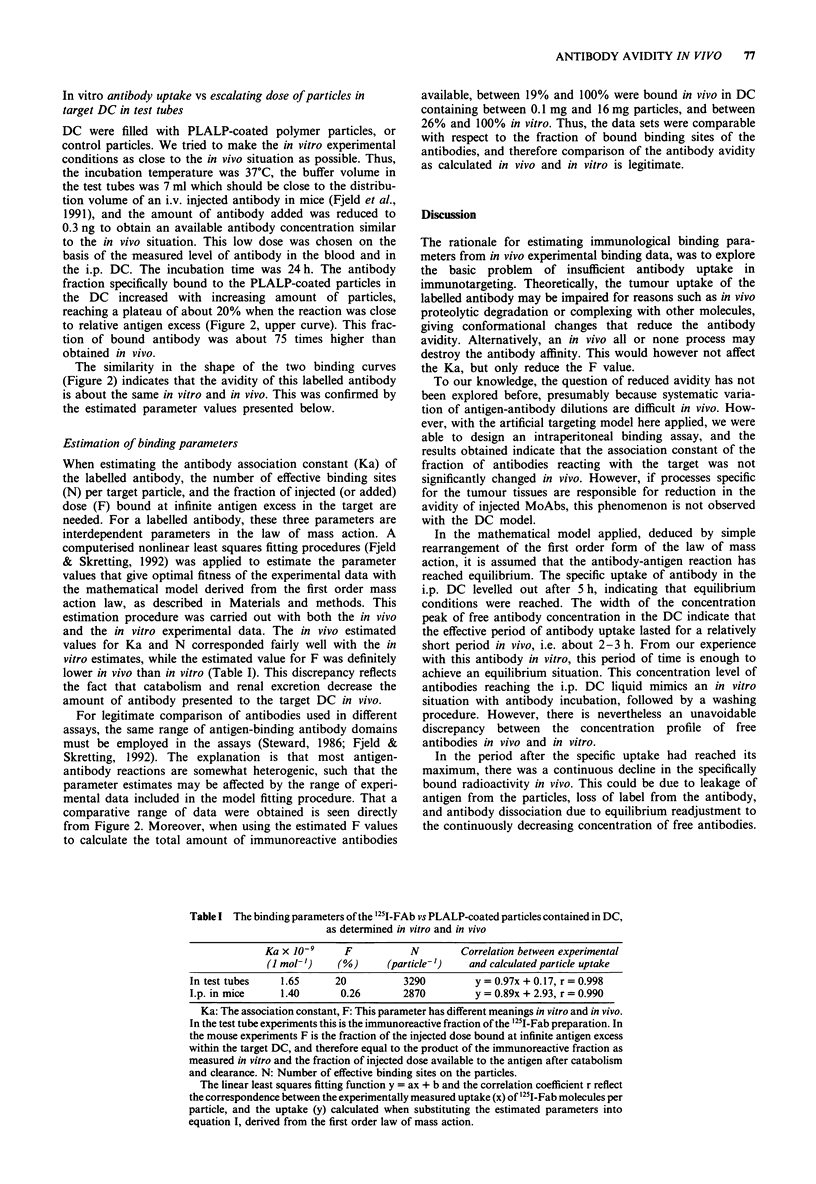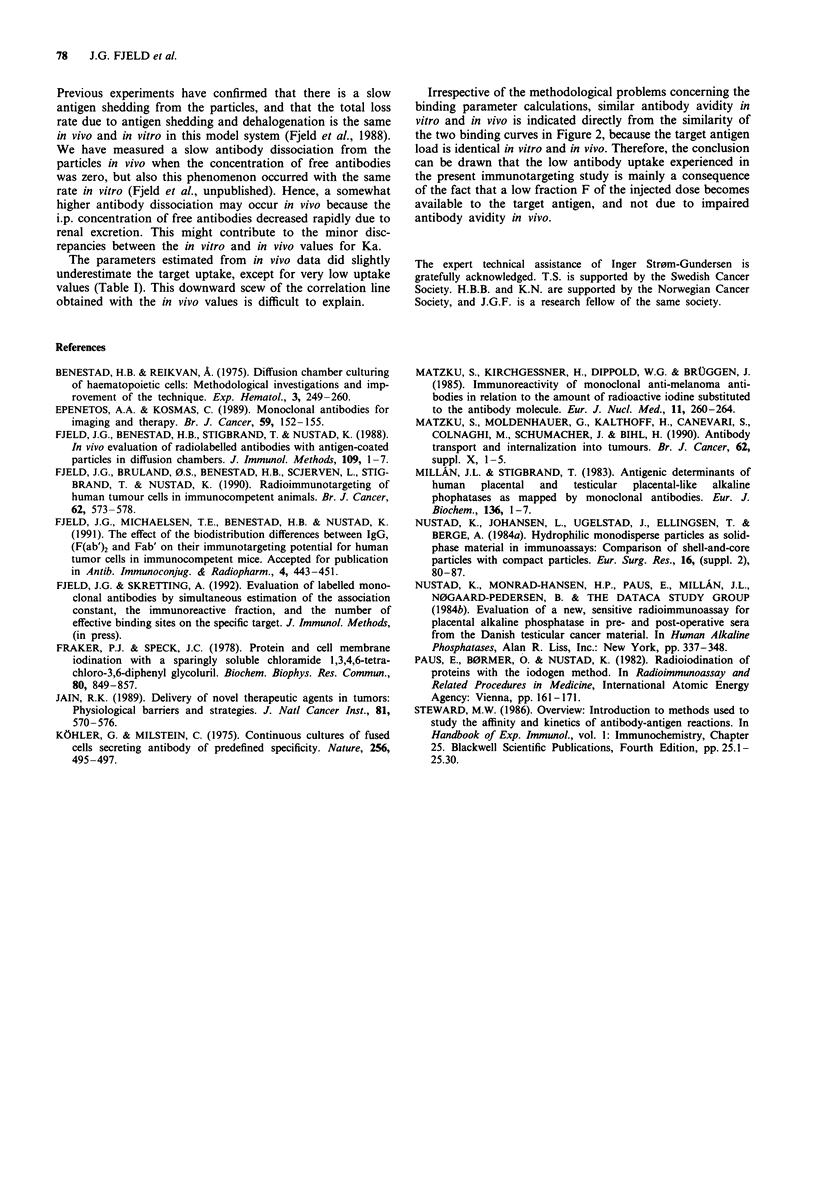# In vivo measurement of the association constant of a radio-labelled monoclonal antibody in experimental immunotargeting.

**DOI:** 10.1038/bjc.1992.219

**Published:** 1992-07

**Authors:** J. G. Fjeld, H. B. Benestad, T. Stigbrand, K. Nustad

**Affiliations:** Central Laboratory, Norwegian Radium Hospital, Montebello, Oslo.

## Abstract

Exploring the fundamental mechanisms behind the low tumour uptake of labelled monoclonal antibodies (MoAbs) during in vivo immunotargeting, experiments were performed to estimate the in vivo value of the association constant (Ka) in an experimental targeting reaction. An artificial tumour model was utilised, based on diffusion chambers (DC) filled with antigen-coated polymer particles, implanted i.p. in normal, immunocompetent mice (NMRI/BOM). The MoAb H7 with specificity for placental alkaline phosphatase (PLALP) was chosen for this experiment. Each mouse carried two DC, one target DC filled with PLALP-coated particles, and a second control DC with the same amount of uncoated particles. The DC contained escalating doses of particles, ranging from 0.1 mg to 16 mg per DC, with groups of 6-12 animals per dose level. The next day after the implantation, a constant dose of 125I-labelled Fab fragments of H7 was injected i.v. in each mouse. The association constant Ka as measured from the binding data obtained in vivo was not significantly different from the value measured in vitro when the same target DC were incubated with the 125I-Fab in test tubes. This indicates that in vivo impairment of the antibody avidity is not the reason why a relatively low tumour uptake is generally experienced in immunotargeting studies.


 © Macmillan Press Ltd., 1992

### *In vivo* measurement of the association constant of a radio-labelled monoclonal antibody in experimental immunotargeting

J.G. Fjeld[1], H.B. Benestad[2], T. Stigbrand[3] & K. Nustad[1]

[1]*Central Laboratory, The Norwegian Radium Hospital, Montebello, N-0310 Oslo, Norway;* [2]*Institute of Basic Sciences, Department of Physiology, University of Oslo, PO Box 1103 Blindern, N-0317 Oslo, Norway;* [3]*Department of Physiological Chemistry, University of Umeå, S-901 87 Umeå, Sweden.*

**Summary** Exploring the fundamental mechanisms behind the low tumour uptake of labelled monoclonal antibodies (MoAbs) during *in vivo* immunotargeting, experiments were performed to estimate the *in vivo* value of the association constant (Ka) in an experimental targeting reaction. An artificial tumour model was utilised, based on diffusion chambers (DC) filled with antigen-coated polymer particles, implanted i.p. in normal, immunocompetent mice (NMRI/BOM). The MoAb H7 with specificity for placental alkaline phosphatase (PLALP) was chosen for this experiment. Each mouse carried two DC, one target DC filled with PLALP-coated particles, and a second control DC with the same amount of uncoated particles. The DC contained escalating doses of particles, ranging from 0.1 mg to 16 mg per DC, with groups of 6–12 animals per dose level. The next day after the implantation, a constant dose of $^{125}$I-labelled Fab fragments of H7 was injected i.v. in each mouse.

The association constant Ka as measured from the binding data obtained *in vivo* was not significantly different from the value measured *in vitro* when the same target DC were incubated with the $^{125}$I-Fab in test tubes. This indicates that *in vivo* impairment of the antibody avidity is not the reason why a relatively low tumour uptake is generally experienced in immunotargeting studies.

With the development of the hybridoma technology for production of monoclonal antibodies (Köhler & Milstein, 1975), there has been an increasing interest in diagnosis and treatment of cancer with labelled antibodies to tumour associated antigens (Epenetos & Kosmas, 1989). However, for various reasons, the tumour uptake of labelled MoAbs is disappointingly low in most targeting studies. Firstly, non-specific binding of antibody in normal tissues is regularly experienced. Secondly, the antibody concentration in the tumour is lowered due to antibody catabolism and clearance. Thirdly, various local mechanisms such as tumour vasculature, blood flow, tumour tissue permeability, and the tumour interstitial pressure hampers the antibody access to the tumour antigen (Jain, 1989; Matzku *et al.*, 1990).

Moreover, as in every immunological reaction, the avidity of the antibody should presumably be a factor of uttermost importance to the tumour uptake. If the avidity is impaired *in vivo*, this would contribute to the low tumour uptake. To be able to explore this question, experimental binding data from *in vivo* targeting reactions where the amounts of antibody and antigen are under control is required. This problem was overcome by applying the artificial tumour model elaborated by us (Fjeld *et al.*, 1988). The model is based on intraperitoneal diffusion chambers (DC) filled with a suspension of antigen-coated polymer particles. Any experimental animal species large enough to carry the DC can be utilised (Fjeld *et al.*, 1990). With this model system it is possible to systematically vary the antigen concentration in the target. Each target has the same size, and measurement of specific antibody uptake is made possible by the antigen-negative control target carried by each animal.

The aim of the present work was to compare the *in vivo* and *in vitro* avidity of a high avidity Fab fragment of the MoAb H7, specific for placental alkaline phosphatase (PLALP) (Millán & Stigbrand, 1983). The *in vivo* immunotargeting is performed with PLALP-coated DC i.p. in normal, immunocompetent mice, while the *in vitro* experiments are carried out with the DC kept in test tubes with buffer, with the labelled antibody added.

Correspondence: J.G. Fjeld, Department of Clinical Chemistry, The National Hospital, N-0027 Oslo, Norway.
Received 25 November 1991; and in revised form 19 March 1992.

### Materials and methods

*Monodisperse polymer particles coated with placental alkaline phosphatase (PLALP)*

Hydrophilic monodisperse shell-and-core polymer particles with diameter 3.3 μm were used (Nustad *et al.*, 1984*a*; Nustad *et al.*, 1984*b*), and the particles were coated with PLALP as previously described (Fjeld *et al.*, 1988), using PLALP isolated from placentae at term (Nustad *et al.*, 1984*b*).

The amount of PLALP on the particle surface was quantified as enzyme activity, and as antigen for its specific antibody H7. By determination of the enzyme activity, using p-dinitrophenyl phosphate as substrate, the amount of particle-bound PLALP was determined to be $32\cdot10^{-10}$ pmol (1900 PLALP molecules) per particle (Fjeld *et al.*, 1988). An estimate of the binding capacity of the particles for the $^{125}$I-labelled Fab fragment of the PLALP-specific MoAb H7 was obtained by mixing a relatively high and constant amount of $^{125}$I-Fab with decreasing number of particles. In dilutions close to antibody excess, the antibody uptake per particle levelled out, and the amount of $^{125}$I-Fab bound at this plateau level was $53\cdot10^{-10}$ pmol (3200 molecules) per particle, or an average of 1.7 $^{125}$I-Fab per PLALP molecule on the particle surface. This corresponds with the dimeric structure of the PLALP molecule.

*Preparation of diffusion chambers (DC)*

DC were constructed as described (Benestad & Reikvam, 1975). In short, the DC consisted of two Millipore GSWP 0.22 μm micropore membranes (Millipore[R] Corp., Bedford, Mass) heat sealed to both sides of a 2 mm thick acrylic plastic ring, outer diameter 13 mm. All chambers were filled with 160 μl particle suspension from a syringe through an orifice in the plastic ring, and closed with a conical plastic plug. The particles were suspended in PBS with 5% normal mouse serum and 200 μg ml$^{-1}$ ampicillin.

*Mice*

Randomly bred female mice (NMRI/BOM), 8–12 weeks of age, were used.

*DC implantation in mice*

The operation was carried out under ether anaesthesia the day before antibody injection. The DC were i.p. implanted through a midline laparotomy, and the wounds were closed with metal clips. Each mouse received two DC, one target DC filled with PLALP-coated polymer particles, and a second control DC with a corresponding number of PLALP-free particles.

*Antibody*

The antibody was a Fab fragment of the MoAb H7 (IgG2a, kappa), specific for the three common allelic variants of PLALP and most types of testis PLALP-like enzymes (Millán & Stigbrand, 1983). Fab fragments were prepared as described earlier (Fjeld *et al.*, 1988).

*Radiolabelling with $^{125}I$*

The Fab fragments were labelled with $^{125}$I, using Iodo-Gen (Pierce, Rockford, Illinois) as oxidant (Fraker & Speck, 1978; Paus *et al.*, 1982). The iodination procedure was carried out the day before i.v. injection.

*Antibody injection*

The $^{125}$I-Fab was diluted in 0.9% NaCl with 0.1% normal mouse serum. The next day after the DC implantation, the mice were injected i.v. (tail vein) with 100 ng $^{125}$I-Fab in 200 µl.

*Harvest of the intraperitoneal DC*

The two DC carried by each animal, i.e. one DC with the antigen-coated particles and a second control DC, were collected from animals killed by ether overdosage. The DC were immediately removed, gently wiped off with a piece of soft paper, and the radioactivity in the whole DC was counted in a multiwell gamma counter. The specific binding to the antigen-coated particles was obtained from the difference in radioactivity between the target and control DC.

*DC incubated* in vitro *in test tubes*

The $^{125}$I-Fab uptake in DC *in vitro* was measured using test tubes with 7 ml PBS with 1% BSA and 0.01% $NaN_3$. Each test tube contained one target DC and a second control DC, $^{125}$I-FAb was added (0.3 ng 50 $\mu l^{-1}$), and the tubes were rotated end over end at 37°C. As in the animal experiments, the target and control DC were gently wiped off with soft paper, the radioactivity in the whole DC were counted, and the specific uptake was measured as the difference between the target and control DC.

*Theoretical background for the* in vivo *parameter estimation*

Presuming that the targeting reaction between the $^{125}$I-Fab and its binding sites on the PLALP-coated particles is a bimolecular and reversible reaction, and that both the reactants have homogeneous binding characteristics, the reaction will obey the first order form of the mass action law. Then, as previously described for labelled MoAb - antigen reactions in general (Fjeld & Skretting, 1992), simple rearrangement of the mass action law gives the following model equation for the reaction at equilibrium:

$$B(i) = (A-(A^2-C)^{0.5})/D \qquad (I)$$

Adaption of equation I to the DC model system for RIT gives the following definitions:

A = $1 + Ka \cdot F \cdot T + Ka \cdot N \cdot M(i)$
C = $4 \cdot Ka^2 \cdot N \cdot F \cdot M(i) \cdot T$
D = $2 \cdot Ka \cdot M(i)$
N = Total number of effective binding sites per polymer particle
B(i) = Number of specifically bound $^{125}$I-Fab molecules per polymer particle within a specified DC
T = The dose of $^{125}$I-Fab injected i.v.
M(i) = Concentration of PLALP-coated polymer particles within a specified DC
F = The maximal fraction of injected dose of $^{125}$I-Fab that is bound to the target particles in the DC, when the concentration of effective binding sites on the PLALP-coated particles is in extreme excess relative to the concentration of the $^{125}$I-Fab molecules available.

Equation I has three unknown parameters Ka, F and N. The constant antibody dose T and the systematically varied concentration of particles M(i) in the target DC are chosen values, whereas B(i) is the experimentally measured specific antibody uptake in the target DC. With three unknown parameters and only one equation, an iterative nonlinear least squares approximation method was used to obtain the optimally fitting binding parameters (Fjeld & Skretting, 1992).

The parameter F is analogous to the immunoreactive fraction of the labelled antibody preparation. To use the expression 'immunoreactive fraction' for the parameter F *in vivo* is however somewhat misleading, because the major reason for non-reactivity *in vivo* is excretion and catabolism of the i.v. injected antibody molecules, before they become available for the target antigen. Only the local concentration of antibody molecules within the DC shall be taken into account in the parameter calculations. Consequently, to be able to estimate antibody binding parameters from data measured *in vivo*, the following problem must be solved: How large a fraction of the injected antibody dose is actually presented to the tumour antigens? The sum of all of the different processes that affect this fraction, such as extravasation, excretion, catabolism, and diffusion across the DC wall, may not be a linear function of the injected dose, and then the parameter F defined above will vary with the injected dose. This was solved by injecting the constant dose T of $^{125}$I-Fab to all the animals. The parameter F should then also have a constant value, except for the biological and random experimental variation from animal to animal.

### Results

*The kinetics of antibody uptake in the i.p. DC*

Immunological binding parameters characterise the antigen-antibody reaction under equilibrium conditions. As an introductory experiment, exploring if our *in vivo* targeting reaction was close to equilibrium, a dynamic study of the target uptake of the $^{125}$I-Fab preparation was carried out. In this experimental set-up the particle concentration in the i.p. target DC and the dose of antibody injected i.v. were kept constant, while the time interval between injection and target harvest was varied (Figure 1).

The antibody concentration in the control DC (Figure 1, upper curve), reflecting the concentration of free antibody within the target, increased rapidly and reached its maximum about 3 h after the i.v. injection. This rapid increase was followed by a period with rapid decrease towards a low background level, giving a relatively sharp peak in the concentration of free, available antibodies within the DC. This indicates a rapid extravasation and a good access to the target for this fragmented antibody, followed by a rapid excretion.

The specific uptake in the target DC (Figure 1, lower curve) did also increase immediately after the injection. The increase rate was however somewhat lower than for the free antibody concentration, and the maximum specific uptake was reached about 5 h after injection. In the following period the amount of specifically bound radioactivity decreased slowly.

From these results, a 24 h interval between injection and tumour harvest was chosen for the consecutive series of

**Figure 1** The kinetics of the nonspecific (*upper curve*) and specific uptake (*lower curve*) of $^{125}$I-Fab fragments of the MoAb H7, when administered i.v. to mice carrying i.p. implanted DC. The DC contained 0.1 mg PLALP-coated particles in 160 µl buffer (target DC), or the same amount of PLALP-free control particles (control DC). Each mouse was implanted i.p. with one target DC and one control DC, and the next day after the operation the mice were injected i.v. (tail vein) with $^{125}$I-Fab (100 ng 200 µl$^{-1}$; 7 × 10$^6$ c.p.m.). The upper curve includes all radioactivity in the control DC, and this curve reflects therefore the variation in i.p. concentration of free $^{125}$I-Fab. The specific uptake in the target DC have been corrected for the uptake in the neighbouring control DC. Results from single DC.

experiments, carried out to obtain binding data for the binding parameter estimation. After 24 h the specific uptake had levelled out, i.e. the reaction had apparently reached equilibrium, and the concentration of free antibodies had reached a low background level.

The signal to noise ratio of the data in Figure 1 is poor. However, the antigen concentration was low. The concentration of particles (0.1 mg 160 µl$^{-1}$) in this study of the kinetics of the antibody uptake was chosen to be in the lower part of the range of particle concentrations planned to be used in the consecutive study carried out to obtain data for calculation of the immunological binding parameters for an *in vivo* immunological reaction. The rationale for choosing this low antigen level in the introductory kinetic study, was that if the targeting reaction with the lowest particle concentration (0.1 mg 160 µl$^{-1}$) in the DC is close to equilibrium, then the reactions between the same dose of antibody and higher particle concentrations will also be close to equilibrium. The DC components, and not the particles, are responsible for the nonspecific binding. The signal to noise ratio in this model system has been explored in a previous work (Fjeld *et al.*, 1988).

### In vivo *antibody uptake* vs *escalating dose of particles in the i.p. target DC*

To obtain targeting data for calculation of immunological binding parameters of the targeting reaction, a constant dose of labelled antibody (100 ng 200 µl$^{-1}$) was injected i.v. in mice carrying i.p. DC with serial dilutions of particles ranging from 0.1 mg 160 µl$^{-1}$ to 16 mg 160 µl$^{-1}$. The specific uptake increased with increasing concentration of particles, reaching a plateau level of about 0.25% of injected antibody dose (Figure 2, lower curve). This represents the fraction of injected dose that is bound when the particle concentration in the DC is in relative excess. Thus, the plateau level is close to the value of the parameter F.

The signal to noise ratio in this experiment increased from a poor level of 1 with 0.1 mg particles, to a ratio of 4 with 16 mg particles.

Exploring the sensitivity in this targeting system, experiments were also carried out with mice carrying DC with extremely low antigen content: 0.03 mg, 0.05 mg and 0.07 mg particles. Each DC gave a specific uptake significantly different from zero, and the uptake results were ranged according to their antigen content, indicating that this is a reliable system for studying antibody-antigen reactions *in vivo*. The explanation probably is that each animal carry a second control DC, reducing the effect of the biological and experimental variation on the specific uptake. These data, representing very low percentages of the injected dose bound, were however not included in the parameter calculation procedure. The mathematical model underlying the parameter estimation, i.e. the first order mass action law, assumes that both the antigen and the antibody have homogeneous binding characteristics. There may be heterogeneity in the avidity of labelled MoAbs (Matzku *et al.*, 1985; Fjeld & Skretting, 1992). Then, results from DC with very low antigen content will be dominated by the antibody molecules with the highest avidity. To reduce this problem when estimating the parameters, the data included in the estimation procedure were restricted to the results from DC with a range of particles between 0.1 mg and 16 mg.

**Figure 2** *In vitro* and *in vivo* binding of $^{125}$I-Fab to PLALP-coated polymer particles in DC. Upper curve: DC kept at 37°C *in vitro* in test tubes with 7 ml buffer for 24 h, rotated end over end. Results from DC containing from 0.1 mg to 16 mg particles. Lower curve: i.p. DC in mice, harvested 24 h after i.v. injection of antibody. The antibody dose was 0.3 ng $^{125}$I-Fab (50 µl) in the test tubes, added at time 0, while 100 ng (200 µl) were administered i.v. (tail vein) in the mice. Results from DC containing from 0.03 mg to 16 mg particles. Median values for three replicates *in vitro*, and six replicates *in vivo*.

### In vitro *antibody uptake* vs *escalating dose of particles in target DC in test tubes*

DC were filled with PLALP-coated polymer particles, or control particles. We tried to make the *in vitro* experimental conditions as close to the *in vivo* situation as possible. Thus, the incubation temperature was 37°C, the buffer volume in the test tubes was 7 ml which should be close to the distribution volume of an i.v. injected antibody in mice (Fjeld *et al.*, 1991), and the amount of antibody added was reduced to 0.3 ng to obtain an available antibody concentration similar to the *in vivo* situation. This low dose was chosen on the basis of the measured level of antibody in the blood and in the i.p. DC. The incubation time was 24 h. The antibody fraction specifically bound to the PLALP-coated particles in the DC increased with increasing amount of particles, reaching a plateau of about 20% when the reaction was close to relative antigen excess (Figure 2, upper curve). This fraction of bound antibody was about 75 times higher than obtained *in vivo*.

The similarity in the shape of the two binding curves (Figure 2) indicates that the avidity of this labelled antibody is about the same *in vitro* and *in vivo*. This was confirmed by the estimated parameter values presented below.

### *Estimation of binding parameters*

When estimating the antibody association constant (Ka) of the labelled antibody, the number of effective binding sites (N) per target particle, and the fraction of injected (or added) dose (F) bound at infinite antigen excess in the target are needed. For a labelled antibody, these three parameters are interdependent parameters in the law of mass action. A computerised nonlinear least squares fitting procedures (Fjeld & Skretting, 1992) was applied to estimate the parameter values that give optimal fitness of the experimental data with the mathematical model derived from the first order mass action law, as described in Materials and methods. This estimation procedure was carried out with both the *in vivo* and the *in vitro* experimental data. The *in vivo* estimated values for Ka and N corresponded fairly well with the *in vitro* estimates, while the estimated value for F was definitely lower *in vivo* than *in vitro* (Table I). This discrepancy reflects the fact that catabolism and renal excretion decrease the amount of antibody presented to the target DC *in vivo*.

For legitimate comparison of antibodies used in different assays, the same range of antigen-binding antibody domains must be employed in the assays (Steward, 1986; Fjeld & Skretting, 1992). The explanation is that most antigen-antibody reactions are somewhat heterogenic, such that the parameter estimates may be affected by the range of experimental data included in the model fitting procedure. That a comparative range of data were obtained is seen directly from Figure 2. Moreover, when using the estimated F values to calculate the total amount of immunoreactive antibodies available, between 19% and 100% were bound *in vivo* in DC containing between 0.1 mg and 16 mg particles, and between 26% and 100% *in vitro*. Thus, the data sets were comparable with respect to the fraction of bound binding sites of the antibodies, and therefore comparison of the antibody avidity as calculated *in vivo* and *in vitro* is legitimate.

### Discussion

The rationale for estimating immunological binding parameters from *in vivo* experimental binding data, was to explore the basic problem of insufficient antibody uptake in immunotargeting. Theoretically, the tumour uptake of the labelled antibody may be impaired for reasons such as *in vivo* proteolytic degradation or complexing with other molecules, giving conformational changes that reduce the antibody avidity. Alternatively, an *in vivo* all or none process may destroy the antibody affinity. This would however not affect the Ka, but only reduce the F value.

To our knowledge, the question of reduced avidity has not been explored before, presumably because systematic variation of antigen-antibody dilutions are difficult *in vivo*. However, with the artificial targeting model here applied, we were able to design an intraperitoneal binding assay, and the results obtained indicate that the association constant of the fraction of antibodies reacting with the target was not significantly changed *in vivo*. However, if processes specific for the tumour tissues are responsible for reduction in the avidity of injected MoAbs, this phenomenon is not observed with the DC model.

In the mathematical model applied, deduced by simple rearrangement of the first order form of the law of mass action, it is assumed that the antibody-antigen reaction has reached equilibrium. The specific uptake of antibody in the i.p. DC levelled out after 5 h, indicating that equilibrium conditions were reached. The width of the concentration peak of free antibody concentration in the DC indicate that the effective period of antibody uptake lasted for a relatively short period *in vivo*, i.e. about 2–3 h. From our experience with this antibody *in vitro*, this period of time is enough to achieve an equilibrium situation. This concentration level of antibodies reaching the i.p. DC liquid mimics an *in vitro* situation with antibody incubation, followed by a washing procedure. However, there is nevertheless an unavoidable discrepancy between the concentration profile of free antibodies *in vivo* and *in vitro*.

In the period after the specific uptake had reached its maximum, there was a continuous decline in the specifically bound radioactivity *in vivo*. This could be due to leakage of antigen from the particles, loss of label from the antibody, and antibody dissociation due to equilibrium readjustment to the continuously decreasing concentration of free antibodies.

**Table I** The binding parameters of the $^{125}$I-FAb *vs* PLALP-coated particles contained in DC, as determined *in vitro* and *in vivo*

| | *Ka* × $10^{-9}$ ($l\ mol^{-1}$) | *F* (%) | *N* ($particle^{-1}$) | *Correlation between experimental and calculated particle uptake* |
|---|---|---|---|---|
| In test tubes | 1.65 | 20 | 3290 | y = 0.97x + 0.17, r = 0.998 |
| I.p. in mice | 1.40 | 0.26 | 2870 | y = 0.89x + 2.93, r = 0.990 |

Ka: The association constant, F: This parameter has different meanings *in vitro* and *in vivo*. In the test tube experiments this is the immunoreactive fraction of the $^{125}$I-Fab preparation. In the mouse experiments F is the fraction of the injected dose bound at infinite antigen excess within the target DC, and therefore equal to the product of the immunoreactive fraction as measured *in vitro* and the fraction of injected dose available to the antigen after catabolism and clearance. N: Number of effective binding sites on the particles.

The linear least squares fitting function y = ax + b and the correlation coefficient r reflect the correspondence between the experimentally measured uptake (x) of $^{125}$I-Fab molecules per particle, and the uptake (y) calculated when substituting the estimated parameters into equation I, derived from the first order law of mass action.

Previous experiments have confirmed that there is a slow antigen shedding from the particles, and that the total loss rate due to antigen shedding and dehalogenation is the same *in vivo* and *in vitro* in this model system (Fjeld *et al.*, 1988). We have measured a slow antibody dissociation from the particles *in vivo* when the concentration of free antibodies was zero, but also this phenomenon occurred with the same rate *in vitro* (Fjeld *et al.*, unpublished). Hence, a somewhat higher antibody dissociation may occur *in vivo* because the i.p. concentration of free antibodies decreased rapidly due to renal excretion. This might contribute to the minor discrepancies between the *in vitro* and *in vivo* values for Ka.

The parameters estimated from *in vivo* data did slightly underestimate the target uptake, except for very low uptake values (Table I). This downward scew of the correlation line obtained with the *in vivo* values is difficult to explain.

Irrespective of the methodological problems concerning the binding parameter calculations, similar antibody avidity *in vitro* and *in vivo* is indicated directly from the similarity of the two binding curves in Figure 2, because the target antigen load is identical *in vitro* and *in vivo*. Therefore, the conclusion can be drawn that the low antibody uptake experienced in the present immunotargeting study is mainly a consequence of the fact that a low fraction F of the injected dose becomes available to the target antigen, and not due to impaired antibody avidity *in vivo*.

The expert technical assistance of Inger Strøm-Gundersen is gratefully acknowledged. T.S. is supported by the Swedish Cancer Society. H.B.B. and K.N. are supported by the Norwegian Cancer Society, and J.G.F. is a research fellow of the same society.

### References

BENESTAD, H.B. & REIKVAN, Å. (1975). Diffusion chamber culturing of haematopoietic cells: Methodological investigations and improvement of the technique. *Exp. Hematol.*, **3**, 249–260.

EPENETOS, A.A. & KOSMAS, C. (1989). Monoclonal antibodies for imaging and therapy. *Br. J. Cancer*, **59**, 152–155.

FJELD, J.G., BENESTAD, H.B., STIGBRAND, T. & NUSTAD, K. (1988). *In vivo* evaluation of radiolabelled antibodies with antigen-coated particles in diffusion chambers. *J. Immunol. Methods*, **109**, 1–7.

FJELD, J.G., BRULAND, ØS., BENESTAD, H.B., SCJERVEN, L., STIGBRAND, T. & NUSTAD, K. (1990). Radioimmunotargeting of human tumour cells in immunocompetent animals. *Br. J. Cancer*, **62**, 573–578.

FJELD, J.G., MICHAELSEN, T.E., BENESTAD, H.B. & NUSTAD, K. (1991). The effect of the biodistribution differences between IgG, $(F(ab')_2$ and Fab' on their immunotargeting potential for human tumor cells in immunocompetent mice. Accepted for publication in *Antib. Immunoconjug. & Radiopharm.*, **4**, 443–451.

FJELD, J.G. & SKRETTING, A. (1992). Evaluation of labelled monoclonal antibodies by simultaneous estimation of the association constant, the immunoreactive fraction, and the number of effective binding sites on the specific target. *J. Immunol. Methods*, (in press).

FRAKER, P.J. & SPECK, J.C. (1978). Protein and cell membrane iodination with a sparingly soluble chloramide 1,3,4,6-tetrachloro-3,6-diphenyl glycoluril. *Biochem. Biophys. Res. Commun.*, **80**, 849–857.

JAIN, R.K. (1989). Delivery of novel therapeutic agents in tumors: Physiological barriers and strategies. *J. Natl Cancer Inst.*, **81**, 570–576.

KÖHLER, G. & MILSTEIN, C. (1975). Continuous cultures of fused cells secreting antibody of predefined specificity. *Nature*, **256**, 495–497.

MATZKU, S., KIRCHGESSNER, H., DIPPOLD, W.G. & BRÜGGEN, J. (1985). Immunoreactivity of monoclonal anti-melanoma antibodies in relation to the amount of radioactive iodine substituted to the antibody molecule. *Eur. J. Nucl. Med.*, **11**, 260–264.

MATZKU, S., MOLDENHAUER, G., KALTHOFF, H., CANEVARI, S., COLNAGHI, M., SCHUMACHER, J. & BIHL, H. (1990). Antibody transport and internalization into tumours. *Br. J. Cancer*, **62**, suppl. X, 1–5.

MILLÁN, J.L. & STIGBRAND, T. (1983). Antigenic determinants of human placental and testicular placental-like alkaline phophatases as mapped by monoclonal antibodies. *Eur. J. Biochem.*, **136**, 1–7.

NUSTAD, K., JOHANSEN, L., UGELSTAD, J., ELLINGSEN, T. & BERGE, A. (1984*a*). Hydrophilic monodisperse particles as solid-phase material in immunoassays: Comparison of shell-and-core particles with compact particles. *Eur. Surg. Res.*, **16**, (suppl. 2), 80–87.

NUSTAD, K., MONRAD-HANSEN, H.P., PAUS, E., MILLÁN, J.L., NØGAARD-PEDERSEN, B. & THE DATACA STUDY GROUP (1984*b*). Evaluation of a new, sensitive radioimmunoassay for placental alkaline phosphatase in pre- and post-operative sera from the Danish testicular cancer material. In *Human Alkaline Phosphatases*, Alan R. Liss, Inc.: New York, pp. 337–348.

PAUS, E., BØRMER, O. & NUSTAD, K. (1982). Radioiodination of proteins with the iodogen method. In *Radioimmunoassay and Related Procedures in Medicine*, International Atomic Energy Agency: Vienna, pp. 161–171.

STEWARD, M.W. (1986). Overview: Introduction to methods used to study the affinity and kinetics of antibody-antigen reactions. In *Handbook of Exp. Immunol.*, vol. 1: Immunochemistry, Chapter 25. Blackwell Scientific Publications, Fourth Edition, pp. 25.1–25.30.